# Standardising monitoring data on drug-related infectious diseases among people who inject drugs in Europe – an update of the European Union Drugs Agency technical protocol, 2024

**DOI:** 10.2807/1560-7917.ES.2025.30.30.2500007

**Published:** 2025-07-31

**Authors:** Ida Sperle, Thomas Seyler, Filippo Pericoli, Erika Duffell, Sharon Hutchinson, Marie Jauffret-Roustide, Martin Kåberg, Anda Ķīvīte-Urtāne, Carole Seguin-Devaux, Vana Sypsa, Ruth Zimmermann

**Affiliations:** 1 Robert Koch Institute (RKI), Berlin, Germany; 2 European Union Drugs Agency (EUDA), Lisbon, Portugal; 3 European Centre for Disease Prevention and Control (ECDC), Stockholm, Sweden; 4 Glasgow Caledonian University, Glasgow, Scotland; 5 Public Health Scotland (PHS), Edinburgh, Scotland; 6 Centre d’étude des mouvements sociaux (Inserm U1276/CNRS UMR 8044/EHESS), Paris, France; 7 Karolinska Institutet (KI), Department of Global Public Health, Stockholm, Sweden; 8 Institute of Public Health, Riga Stradins University, Riga, Latvia; 9 Luxembourg Institute of Health, Strassen, Luxembourg; 10 Medical School, National and Kapodistrian University of Athens, Athens, Greece

**Keywords:** PWID, DRID, hepatitis, HIV, hepatitis B, hepatitis C, monitoring, protocol, EUDA

## Abstract

Drug-related infectious diseases (DRID), such as HIV, hepatitis B virus (HBV) and hepatitis C virus (HCV), contribute to high morbidity and mortality among people who inject drugs (PWID). The European Union Drugs Agency (EUDA) is responsible for monitoring DRID and related behaviours for PWID in Europe. We updated the EUDA DRID technical protocol which covers all steps from planning to data analysis needed for a survey among PWID. Drug-related infectious disease-specific indicators were revised. To enable a more effective monitoring of the current epidemiological situation, we added specific core indicators, such as prevalence of viraemic HBV, HCV and HIV care cascades and harm reduction-related indicators. HIV pre-exposure prophylaxis and take-home naloxone were added as optional indicators. The process was informed by a European working group, who shared best-practice examples of (repeated) cross-sectional and cohort surveys using different methods in various settings. To reach the World Health Organization’s goal of ending HIV and the viral hepatitis epidemics, comprehensive DRID monitoring among the disproportionately affected PWID population is needed.

## Background

The 2022–2030 World Health Organization (WHO) Global Health Sector Strategy (GHSS) suggests an integrated approach towards its goal of ending HIV and the epidemics of viral hepatitis B and C and sexually transmitted infections (STI) [1], and emphasises the importance of focusing on the populations most affected [1]. The WHO has also published consolidated guidelines on HIV, viral hepatitis and STI prevention, diagnosis, treatment and care for key populations, including people who inject drugs (PWID) [2]. An integrated approach is particularly important for PWID who are disproportionately affected by HIV, hepatitis B virus (HBV) and hepatitis C virus (HCV) infections.

These drug-related infectious diseases (DRID) contribute substantially to morbidity and mortality among PWID. A recent study from the European Union (EU)/European Economic Area (EEA) estimated chronic HCV prevalence to be 0.5% (95% credible interval (CrI): 0.5–0.6), and at least 35.8% (95% CrI: 33.1–38.6) of the overall estimate was associated with injecting drug use [3]. In 2021, an estimated 14.8 million people were injecting drugs globally [4], and an estimated 540,000 (95% Crl: 415,000–704,000) in the EU and Norway [5]. Despite the availability of effective HBV, HCV and HIV prevention measures and treatment options, an estimated 17.8%, 52.3% and 9.1% were infected with HIV, HCV (anti-HCV) and HBV (hepatitis B surface antigen (HBsAg)) globally and 4.5%, 53.2% and 3.2% in western Europe, respectively [6].

Harm reduction programmes, including needle and syringe programmes (NSP) and opioid agonist treatment (OAT) can cost-effectively reduce DRID transmission [7], but their implementation and accessibility within the EU varies [8].

The mission of the European Union Drugs Agency (EUDA) (formally established in 2024 replacing the European Monitoring Centre for Drugs and Drug Addiction (EMCDDA)) is to strengthen EU preparedness on drug-related challenges. Since its inception in 1993, the agency has been monitoring DRID, drug-related harms and preventive measures for PWID in close collaboration with the European Centre for Disease Prevention and Control (ECDC).

To support EU/EEA countries on drug-related challenges, the EUDA has developed guidelines [9] which include epidemiological indicators. Some European countries have performed bio-behavioural studies among PWID that provide reliable data on DRID [10,11], but data gaps and challenges remain. Sampling and recruitment of PWID can be difficult, particularly over time. Moreover, a lack of resources, but also lack of political attention, make it difficult to initiate surveys and ensure sustainability. These challenges contribute to the low completeness of DRID indicators. Only six of 29 countries reported recent data on the prevalence of viraemic HCV infections among PWID [12], an indicator that reflects epidemic and service needs [12].

Updates to the DRID guidance, published in 2013 [9], were necessitated by several developments, including the GHSS targets, the increasing need for viraemic HCV prevalence data due to the rising number of individuals treated with direct-acting antivirals (DAA) and cured and new preventive and diagnostic options.

The aims of the updated EUDA DRID technical protocol were to reflect new developments in DRID since 2013 by updating the existing epidemiological indicators [9] and to simplify the methodological steps to assist EU/EEA countries in data collection. In this perspective, we report on all EUDA epidemiological indicators and highlight in detail those that are new or revised with selected best-practice examples. Further best-practice examples are published in a compilation [10].

## Monitoring drug related infectious diseases in Europe

### Technical protocol

The DRID technical protocol was updated through a collaborative project involving the EUDA, ECDC, two consultants and a DRID working group formed from members of the DRID network [13]. Input was provided and decisions were taken during three consensus meetings. We based the protocol update on a synthesis of evidence from surveys collecting DRID indicators among PWID (here we focus on drugs for psychoactive effects and not those who use drugs to enhance image and performance e.g. anabolic androgenic steroids or peptide hormones) in Europe. We categorised the indicators as core, recommended and optional [13]. We identified surveys from France, Germany, Greece, Luxembourg, Scotland and Sweden, which have successfully collected and reported DRID data, and included them as best-practice examples [10].

### Epidemiological indicators

The epidemiological indicators were structured into four thematic areas: (i) burden and impact, (ii) risk factors, (iii) prevention, and (iv) continuum of care (CoC). Data for all indicators should be collected through biological samples and/or questionnaires in cross-sectional or cohort studies. The denominator for calculating the indicators is the total study population unless otherwise stated.

### Burden and impact indicators

Nine indicators (four core) were defined for collecting data on the burden and impact of HBV, HCV and HIV, including prevalence of chronic/viraemic HBV, HCV and HIV (Table 1). Two new indicators were added (prevalence of viraemic HCV infection, incidence of HCV reinfection). Prevalence data offer important context and serve as a denominator for monitoring CoC. Monitoring prevalence and incidence over time provides useful information to assess the impact of interventions.

**Table 1:** Epidemiological indicators for burden and impact, updated European Union Drugs Agency drug-related infectious diseases technical protocol, 2024

Indicator	Indicator category	Definition	Numerator	Denominator
Prevalence of HBV infection *(unchanged)*	Core	Proportion of PWID with active HBV infection (HBsAg positive)	Number of PWID who tested HBsAg-positive	Number of PWID tested within the study (total population)
Prevalence of viraemic HCV infection *(unchanged)*	Core	Proportion of PWID with active HCV infection (HCV RNA positive or HCV cAg positive)	Number of PWID who tested positive for HCV RNA or HCVcAg	Number of PWID tested within the study (total population)
Prevalence of ever HCV infected *(unchanged)*	Core	Proportion of PWID with positive anti-HCV	Number of PWID who tested anti-HCV positive	Number of PWID tested within the study (total population)
Prevalence of viraemic HCV infection among ever infected ***(new)***	Optional	Prevalence of viraemic HCV infection over those ever infected	Number of PWID who tested positive for HCV RNA / HCVcAg	Number of PWID who tested positive for anti-HCV
Proportion of recent/acute HCV infection *(unchanged)*	Optional	Proportion of anti-HCV negative and HCV RNA/cAg positive participants as a proxy for incidence	Number of PWID who tested anti-HCV negative and HCV RNA/cAg positive	Number of PWID tested within the study (total population)
Prevalence of HIV *(unchanged)*	Core	Proportion of PWID living with HIV infection	Number of PWID who tested HIV positive (confirmed)	Number of PWID tested within the study (total population)
Incidence of HCV infection *(unchanged)*	Optional	Incidence rate of new infections with HCV (HCV RNA/cAg-positive)	Total number of new infections with HCV (HCV RNA/cAg-positive)	Total population minus people living with hepatitis C (HCV RNA/cAg-positive) (person time at risk)
Incidence of HCV re-infection ***(new)***	Optional	Incidence rate of re-infections with HCV (HCV RNA/cAg)	Total number of new infections with HCV (HCV RNA/cAg) among people who have cleared the infection following DAA treatment	People who have cleared the infection following DAA treatment (person-time at risk)
Incidence of HIV infection *(unchanged)*	Optional	Incidence rate of new HIV infections	Total number of new infections with HIV (seroconversion)	Total population minus people living with HIV (person-time at risk)

DAA: direct-acting antivirals; HBsAg: hepatitis B surface antigen; HBV: hepatitis B virus; HCV: hepatitis C virus; HCVcAg: hepatitis C core antigen; PWID: people who inject drugs.

Text in bold refers indicators that have changed in the updated version.

### Prevalence of viraemic hepatitis C virus infection among ever infected

To calculate this new indicator, the number of PWID with viraemic HCV infection (HCV RNA or core antigen (cAg) positive) is used as the numerator, and the denominator is PWID who tested positive for anti-HCV. This new indicator complements the core indicator on prevalence of viraemic HCV infection in the total population. Prevalence among those ever infected allows for more accurate comparison across countries, recognising that anti-HCV varies geographically [14].

Data on viraemic HCV infection can be difficult to collect. In the latest data reported to EUDA, only six countries provided this information [5], while data on anti-HCV prevalence was reported by 20 countries [5]. With an increasing roll-out of DAAs and higher numbers of individuals cured, information on anti-HCV prevalence provides limited understanding of the current HCV burden.

### Incidence of hepatitis C virus reinfection

To calculate this new indicator, the total number of new infections with HCV (HCV RNA/cAg) among people who have previously cleared the infection is used. Monitoring incidence of HCV reinfection is increasingly important due to the scale-up of DAAs, but challenged by the need for observational study designs. Generally, HCV incidence data are useful to acknowledge the dynamics of the epidemic, particularly among PWID with high risk of infection and reinfection. The HCV incidence indicator is defined as the total number of new infections with HCV (HCV RNA/cAg) in the total population (excluding those already living with HCV (HCV RNA/cAg) (total person-time at risk)).

Typically, incidence is estimated through cohort studies, however, these are time-consuming and costly. Other methods include estimates from repeated cross-sectional studies, including modelling, as recommended by the WHO [15], or by applying assumptions of the time of infection, as was done in one study from Greece [10]. Direct incidence estimates can also be generated by using biological markers of recent infection in cross-sectional studies (either anti-HCV avidity testing or HCV RNA testing of anti-HCV negative) [16], but these methods are limited due to large variations in the window period. Another incidence proxy is anti-HCV prevalence among PWID under 25 years of age or among new injectors (see: Proportion of new injectors). As a proxy measure of impact, we included viraemic prevalence among PWID (over time), which is easier to measure through repeated epidemiological surveys compared with specific incidence studies.

Studies that are able to follow persons prospectively and provide repeated testing at regular intervals are considered best practice for measuring incidence [10]. In Stockholm, Sweden, a registry-based study reports on HCV incidence by testing all participants attending an NSP upon enrolment, and every 3–6 months. A new HCV infection is defined as an anti-HCV-negative case at enrolment who became anti-HCV positive during follow-up. A reinfection is defined as an anti-HCV-positive but HCV RNA-negative case who became HCV RNA-positive during follow-up [10].

### Risk factor indicators

Eight indicators (one core) are defined for collecting behavioural data on risk factors. One new indicator has been added (experience with discrimination when accessing healthcare), and two have been revised (Table 2).

**Table 2:** Epidemiological indicators for risk factors, updated European Union Drugs Agency drug-related infectious diseases technical protocol, 2024

Indicator	Indicator category	Definition	Numerator	Denominator
Prevalence of injecting with needles/syringes that were already used by others *(unchanged)*	Core	Proportion of PWID injecting with used needles/syringes in the last 30 days	Number of PWID reporting injecting with used needles/syringes in the last 30 days	Number of PWID included in the study who answered the question on using used needles/syringes
Prevalence of using other paraphernalia already used by others *(unchanged)*	Recommended	Proportion of PWID sharing any used injecting paraphernalia in the last 30 days other than needles/syringes (using together, receiving or passing on)	Number of PWID reporting sharing used injecting paraphernalia in the last 30 days other than needles/syringes (together, receiving or passing on)	Number of PWID included in the study who answered the question on sharing used injecting paraphernalia
Frequency of injection in the last 30 days *(unchanged)*	Recommended	Mean/median number of injections in the last 30 days is calculated using the mean/median number of days with IDU in the last 30 days multiplied by mean/median number of injections on average consuming day in last 30 days	Number of days with injecting drug use in the last 30 days Number of injections on an average consuming day in the last 30 days	Number of PWID who reported injecting in the last 30 days who answered both questions on number of injecting drug use days and number of injections
Proportion of new injectors ***(revised)***	Recommended	Proportion of PWID who started injecting in the last 2 years is one category of number of years since first injection. This number is calculated by subtracting the age at first injection from the current age	Number of PWID who started injecting in the last 2 years	Number of PWID who answered the question on age (years) at the time of the study and age (years) at first injection
Prevalence of injecting drug use, by substance ***(revised)***	Recommended	Proportion of PWID injecting in the last 30 days, by substance (heroin, methadone, buprenorphine, fentanyl and derivatives, cocaine, crack cocaine, methamphetamine, amphetamine, synthetic cathinones, others)	Number of PWID injecting in the last 30 days, by substance	Number of PWID included in the study who answered the question on substances injected
Prevalence of past Imprisonment *(unchanged)*	Recommended	Proportion of PWID who report having ever been in prison	Number of PWID with history of imprisonment	Number of PWID included in the study who answered the question on past imprisonment
Prevalence of homelessness in the last 12 months or currently (*unchanged*)	Recommended	Proportion of PWID who lived without a steady home, on the streets or temporarily in a hostel or shelter, any time in the last 12 months	Number of PWID who experienced homelessness in the last 12 months	Number of PWID included in the study who answered the question on homelessness
Experience with discrimination when accessing healthcare, the last 12 months ***(new)***	Recommended	Proportion of PWID who have experienced discrimination when accessing healthcare the last 12 months	Number of PWID who reported experience of discrimination when accessing healthcare the last 12 months	Number of PWID included in the study who answered the question on discrimination

IDU: injecting drug use; PWID: people who inject drugs.

Text in bold refers indicators that have changed in the updated version.

### Proportion of new injectors

To calculate this revised indicator, the number of PWID who started injecting the last 2 years is used, derived from years since first injection. This subgroup is identified by subtracting age at first injection from age at survey participation.

New injectors are at increased risk of HCV infection, with an estimated higher incidence compared with long-term injectors [17]. Anti-HCV prevalence over time within this group can be used as an important proxy for incidence as prevalence will mirror new transmissions.

### Prevalence of injecting drug use, by substance

This revised indicator is calculated from the number of PWID injecting drugs in the last 30 days by self-reported substance use. Knowledge of substance use and changing injection patterns is important to guide prevention strategies. The injection of stimulants has been linked to increased risk of DRID through increased frequency of use and sharing of injecting paraphernalia [2].

### Experience with discrimination when accessing healthcare

This new indicator is calculated from the number of PWID who reported experiencing discrimination in the last 12 months. Studies report that PWID experience stigma and discrimination which affects both their mental and physical health [18] and constitutes important barriers for prevention and treatment services. Stigma and discrimination have many components, but as an exploratory first step into understanding their impact on DRID, we included one indicator on experienced discrimination related to healthcare.

In Scotland, the Needle Exchange and Syringe Initiative (NESI) [10] collects data on self-reported discrimination by asking if the person has experienced discrimination, has been prevented from doing something or been hassled the last 6 months due to different circumstances, including injecting drug use, HIV and HCV status.

### Prevention indicators

Six indicators (two core) are defined for collecting data on prevention. Three new indicators have been added (Table 3).

**Table 3:** Epidemiological indicators for prevention, updated European Union Drugs Agency drug-related infectious diseases technical protocol, 2024

Indicator	Indicator category	Definition	Numerator	Denominator
Needle-syringe distribution ***(new)***	Core	Average number of sterile needles–syringes received per person who injects drugs in the last 12 months	Number of sterile needles/syringes received from NSP per PWID in the last 30 days	NA For this indicator we compute an average over all responses and multiply by 12 to get a yearly estimate
OAT coverage *(unchanged)*	Core	Proportion of PWID consuming opioids currently receiving medically-prescribed OAT	Number of PWID who receive OAT at the time of the study	Number of PWID consuming opioids or on OST included in the study who answered the question on OAT
HBV vaccination coverage *(****revised****)*	Recommended	Proportion of PWID being vaccinated against HBV	Number of PWID who have received a hepatitis B vaccine	Number of PWID included in the study with information on HBV vaccination
Condom use *(unchanged)*	Recommended	Proportion of PWID reporting the use of a condom at last sexual intercourse	Number of PWID reporting the use of a condom at last sexual intercourse	Number of PWID included in the study who answered condom use
PrEP use ***(new)***	Recommended	Proportion of PWID receiving PrEP	Number of PWID receiving PrEP at the time of the study	Number of PWID included in the study who answered the question on PrEP
Naloxone coverage ***(new)***	Recommended	Proportion of PWID carrying naloxone	Number of PWID who are carrying Naloxone at the time of the study	Number of PWID included in the study who answered the question on naloxone

HBV: hepatitis B virus; NA: not applicable; NSP: needle and syringe programmes; OAT: opioid agonist treatment; PrEP: HIV pre-exposure prophylaxis; PWID: people who inject drugs.

Text in bold refers indicators that have changed in the updated version.

### Needle-syringe distribution

To calculate this new indicator, the number of sterile needles/syringes received from NSP per PWID in the last 30 days is used. Needle and syringe programmes are a cost-saving intervention and have been implemented in all EU countries, but with varying coverage [5].

### Hepatitis B virus vaccination coverage

To calculate this revised indicator (recategorised from core to recommended), the number of PWID being vaccinated against HBV is used.

To determine HBV vaccination coverage, documented vaccination status is needed. Record linkage is an option where data from different databases, vaccination cards or registries are combined. Using self-reported HBV vaccination status is also possible, although this can be affected by recall bias [10]. A German cross-sectional study (acronym: DRUCK study) [10] found a discrepancy between self-reported HBV vaccination status and measured anti-HBs in blood samples [10]. While testing of anti-HBs may seem like a more robust approach, it requires more resources and antibodies wane over time. Moreover, there is a low sensitivity of anti-HBs when tested from dried blood spots [10] and we recommend only to use the titre if tested from venous blood.

In many countries, HBV vaccination has been implemented as part of the childhood vaccination programme [19]. This means vaccine coverage will depend on the age of participants, time of implementation and coverage of childhood vaccination programme and the proportion of people who migrated from countries with a different vaccination policy.

### HIV pre-exposure prophylaxis use

To calculate this new indicator, the number of PWID receiving HIV pre-exposure prophylaxis (PrEP) at the time of the study is used.

It is recommended that PrEP be integrated into HIV prevention services and offered to people at high risk of HIV [20]. While PrEP has been implemented in many countries and has been proven important in preventing HIV among men who have sex with men, studies suggest that uptake and access among PWID is low [20]. This indicates the need to raise more awareness around PrEP, especially for PWID with sexual risks. Information on PrEP use will inform current use and indicate gaps in access to PrEP.

### Naloxone coverage

To calculate this new indicator, the number of PWID carrying naloxone at the time of the study is used.

Harm reduction programmes in many countries have shown that providing naloxone, along with training on its use and on resuscitation, can significantly reduce opioid overdose deaths. This is especially important for people leaving prison who face high overdose rates in the first 4 weeks after release [21]. Epidemiological DRID-focused studies should also be used as an opportunity to assess country-level preparedness to public health threats such as synthetic opioids.

### Continuum of care indicators

Fifteen indicators (eight core) are defined for collecting CoC data, including five new indicators (Table 4). The term CoC is constructed around a public health model including all the steps a person goes through from diagnosis to viral suppression and clinical follow up. This is helpful in highlighting CoC pitfalls and allows us to assess each step from infection to care within certain subpopulations.

**Table 4:** Epidemiological indicators for continuum of care, updated European Union Drugs Agency drug-related infectious diseases technical protocol, 2024

Indicator	Indicator category	Definition	Numerator	Denominator
Testing (HIV) *(unchanged)*	Core	Proportion of PWID who have been tested for HIV the last 12 months (not taking into account tests done within the study and excluding those with a known diagnosis of HIV)	Number of PWID reporting an HIV test in the last 12 months (not taking into account tests done within the study and excluding those with a known diagnosis of HIV)	Number of PWID included in the study who answered the question on HIV testing (excluding those with a known diagnosis of HIV)
Diagnosis (HIV) *(unchanged)*	Core	Proportion of PWID living with HIV who know their status	Number of PWID tested positive for HIV in the study who were aware of their HIV-positive status	Number of HIV-positive PWID included in the study who answered the question on HIV status
Treatment (HIV) *(unchanged)*	Core	Proportion of PWID diagnosed with HIV receiving antiretroviral therapy	Number of PWID who were (already) diagnosed with HIV and are currently receiving ART	Number of PWID included in the study who were (already) diagnosed with HIV with information on ART
Viral suppression (HIV) ***(new)***	Recommended	Proportion of PWID living with HIV, and who are on treatment, achieving viral load suppression	Number of PWID who are receiving ART and currently virally suppressed	Number of PWID who were (already) diagnosed with HIV and are receiving ART
Testing (HBV) *(unchanged)*	Recommended	Proportion of PWID who have been tested for HBsAg the last 12 months (not taking into account tests done within the study and excluding those with a known diagnosis of HBV)	Number of PWID reporting an HBV test in the last 12 months (not taking into account tests done within the study and excluding those with a known diagnosis of HBV)	Number of PWID included in the study who answered the question on HBV testing (excluding those with a known diagnosis of HBV)
Testing (HDV) *(****new****)*	Optional	Proportion of PWID who have been tested positive for HBV who have also been tested for anti-HDV the last 12 months	Number of PWID reporting an HDV test in the last 12 months (not taking into account tests done within the study) and excluding those that are HBV-negative	Number of PWID included in the study who answered the question on HDV testing (excluding those with negative HBV test)
Diagnosis (HBV) – ever *(unchanged)*	Optional	Proportion of PWID living with HBV who have ever been diagnosed with HBsAg (who were aware of their infection)	Number of PWID who tested HBsAg-positive who have been diagnosed with HBV (self-reported or with record of past diagnosis)	Number of HBsAg-positive PWID included in the study who answered the question on HBV status
Treatment (HBV) *(unchanged)*	Optional	Proportion of PWID diagnosed with HBV infection receiving HBV treatment	Number of PWID who tested HBsAg-positive who are currently receiving treatment (self-reported or with record of treatment)	Number of PWID included in the study who were (already) diagnosed with HBV with information on HBV treatment
Viral suppression (HBV) ***(new)***	Optional	Proportion of patients with active HBV infection on treatment in whom HBV viral load is suppressed	Number of patients with HBV infection on treatment who have a suppressed viral load (HBV DNA not detectable), based on viral load measurement in the past 12 months	Number of patients with HBV infection on treatment and assessed for viral load in the past 12 months
Testing (HCV) *(unchanged)*	Core	Proportion of PWID who have been tested for HCV the last 12 months (not taking into account tests done within the study)	Number of PWID reporting an HCV test in the last 12 months (not taking into account tests done within the study)	Number of PWID included in the study who answered the question on HCV testing
Diagnosis (HCV) – ever *(unchanged)*	Core	Proportion of anti-HCV-positive and/or HCV-RNA-positive PWID who have ever been diagnosed with viraemic HCV infection	Number of anti-HCV-positive and/or HCV-RNA-positive PWID who have ever been diagnosed with active HCV infection (self-reported or with record of past diagnosis)	Number of anti-HCV-positive and/or HCV-RNA-positive PWID included in the study who answered the question on diagnosis of active HCV infection (ever) (or with available records)
Diagnosis (HCV) – last 12 months *(unchanged)*	Core	Proportion of anti-HCV-positive and/or HCV-RNA-positive PWID who have been diagnosed with viraemic HCV infection in the last 12 months	Number of anti-HCV-positive and/or HCV-RNA-positive PWID who had a diagnosis of HCV infection in the last 12 months (self-reported or with record of diagnosis in the last 12 months)	Number of anti-HCV-positive and/or HCV-RNA-positive PWID included in the study who answered the question on diagnosis of active HCV infection (or with available records)
Treatment (HCV) – ever *(unchanged)*	Core	Proportion of anti-HCV-positive and/or HCV-RNA-positive PWID who have ever received HCV antiviral treatment	Number of anti-HCV-positive and/or HCV-RNA-positive PWID who have ever received HCV antiviral treatment (self-reported or with record of treatment)	Number of anti-HCV-positive and/or HCV-RNA-positive PWID included in the study who answered the question on HCV antiviral treatment (or with available records)
Treatment (HCV) - last 12 months ***(new)***	Core	Proportion of anti-HCV-positive and/or HCV-RNA-positive PWID who initiated HCV antiviral treatment in the last 12 months	Number of anti-HCV-positive and/or HCV-RNA-positive PWID who initiated HCV antiviral treatment in the last 12 months (self-reported or with record of treatment initiation in the last 12 months)	Number of anti-HCV-positive and/or HCV-RNA-positive PWID included in the study who answered the question on HCV antiviral treatment (or with available records)
Sustained virological response (HCV) ***(new)***	Recommended	Proportion of patients with hepatitis C cured among those who completed treatment	Number of patients who completed hepatitis C treatment and had a sustained virological response based on viral load measurement 12–24 weeks after the end of treatment (in the past 12 months)	Number of patients who completed hepatitis C treatment and were assessed for sustained virological response 12–24 weeks after the end of treatment (in the past 12 months)

ART: antiretroviral therapy; HBsAg: hepatitis B surface antigen; HBV: hepatitis B virus; HCV: hepatitis C virus; HDV: hepatitis D virus; PWID: people who inject drugs.

Text in bold refers indicators that have changed in the updated version.

Due to the differences between DRIDs and the subsequent differences in testing and treatment, the continuum for HCV is different from those for HBV and HIV. All CoC indicators are therefore defined similarly for HBV and HIV, but differently for HCV as people who have been diagnosed, treated and cured for HCV are no longer in need of HCV treatment. We suggest they can be monitored as separate and independently assessed indicators. The method in which CoC data are collected also depends on survey design, where, for example in cross-sectional studies, the only option is to combine testing data with self-reported survey data obtained through data collection. While all best practice examples [10] can provide at least some of the CoC indicators, the registry-based cohort study from Stockholm allows for monitoring the full CoC [10].

### Diagnosis, testing (hepatitis D virus)

To calculate this new indicator, the number of PWID who have tested positive for HBV and who have also been tested for hepatitis D virus (HDV) is used. This clinical indicator is important and addresses a current knowledge gap in the EU/EEA as HBV and HDV coinfection can result in a more rapid progression of liver fibrosis and liver cancer than HBV infection alone.

### Treatment (hepatitis C virus) in the last 12 months

To calculate this new core indicator, the proportion of HCV-positive PWID (anti-HCV and/or HCV RNA) who initiated treatment in the last 12 months is used.

This new indicator was added since for HCV, the distinction between ever treated and treated the last 12 months is important. This indicator can be obtained either from self-reported information or from cohort or registry-based data. When asking about the last 12 months, a current picture of the treatment uptake will be seen.

National CoC data are often not available as either treatment registries or information regarding injecting drug use are missing. As a result, surveys among PWID are important in providing these data to monitor CoC indicators.

### Viral suppression of hepatitis B virus, hepatitis C virus and HIV

For the three new indicators on viral suppression, a distinction is needed for HBV and HIV vs HCV. For HBV and HIV, the indicators are calculated from the number of PWID who are receiving HBV treatment or antiretroviral therapy (ART), with suppressed viral load the past 12 months. For HCV, the indicator is calculated from the number of PWID with sustained virological response based on viral load measurement after treatment completion (according to national standards), which is especially relevant for monitoring after DAA treatment.

## Conclusion

The updated EUDA DRID technical protocol aims to contribute to improved and up-to-date data on DRID in the EU/EEA by assisting countries in conducting high-quality surveys among PWID. However, heterogeneity in the EU/EEA in terms of DRID, populations and context requires pragmatic and adaptable guidance. While there are core indicators and recommended steps, these may need to be adapted according to local needs and context. Therefore, further reading and examples in the updated guidance are meant to serve as inspiration. The division of indicators into core, recommended and optional is a way to guide priority-setting, if resources and time are scarce.

Well-designed epidemiological bio-behavioural surveys are needed to collect information on DRID, including the complex relationship between often mutually reenforcing risk factors, and the impact of prevention and control measures.

Many developments are now reflected in the updated technical protocol, and best-practice examples across Europe highlight creative ways on how to collect data to monitor DRID. A better understanding of the epidemiological situation will highlight gaps and help identify focus areas and specific PWID subpopulations who need improved access to prevention and treatment measures. Data for action is needed, and with a people-centred approach the WHO goals of ending HIV and viral hepatitis epidemics in the EU/EEA may still be within reach for PWID.

## Data Availability

Not applicable.
